# Supplementary figures and images for: Impact of the cost-of-living crisis on the nature of attempts to stop smoking and to reduce alcohol consumption in Great Britain: A representative population survey, 2021–2022
Source: PLoS One. 2023 May 23;18(5):e0286183. doi: 10.1371/journal.pone.0286183 (PMC10204963; doi:10.1371/journal.pone.0286183)

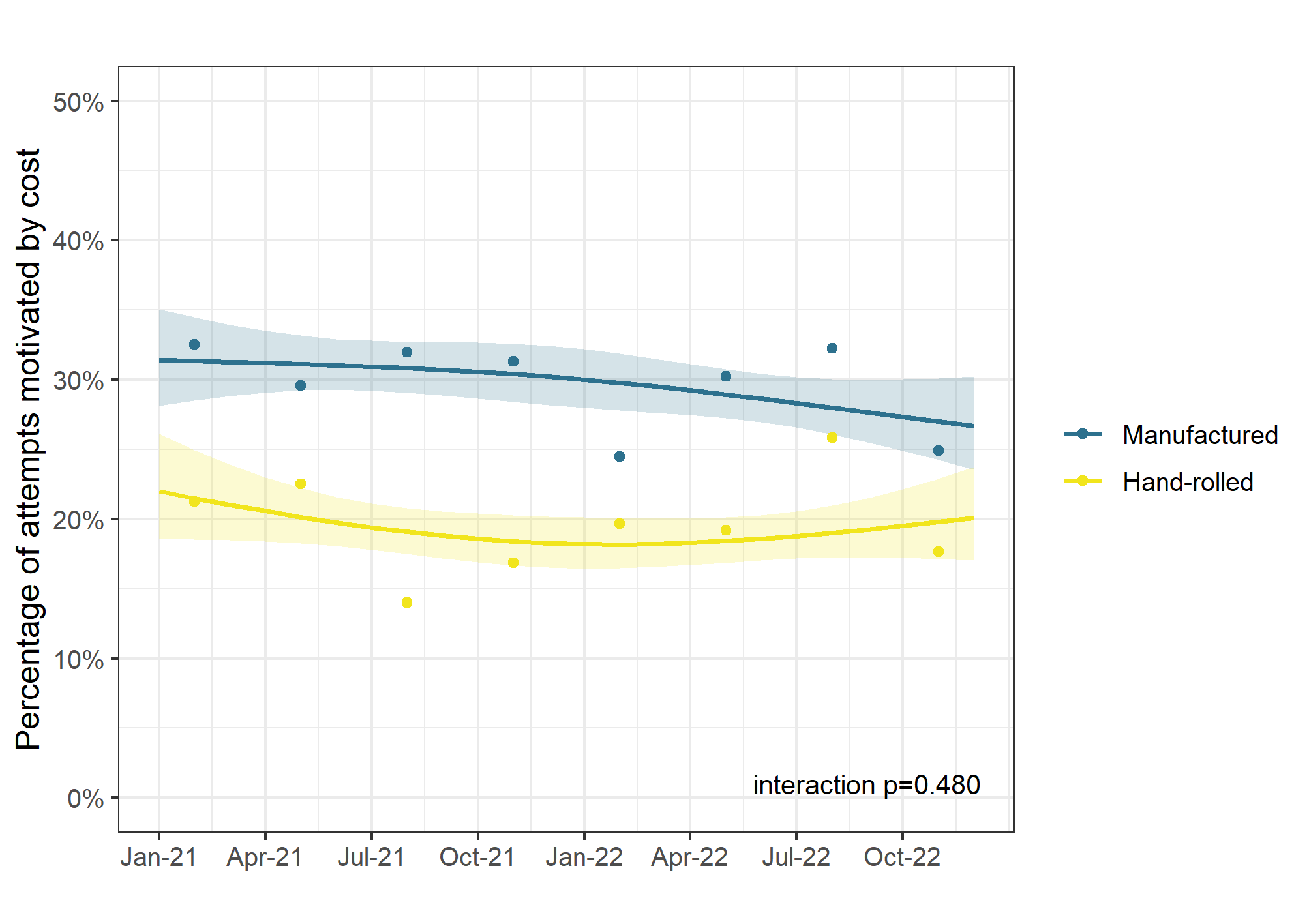

Supplement: S1 Fig — Lines represent modelled weighted prevalence by survey month, modelled non-linearly using restricted cubic splines (three knots), adjusting for covariates. Shaded bands represent standard errors. Points represent raw weighted prevalence by quarter. P-values are for the interaction between survey month and main type of cigarettes smoked. (TIFF) [file pone.0286183.s001.tiff]

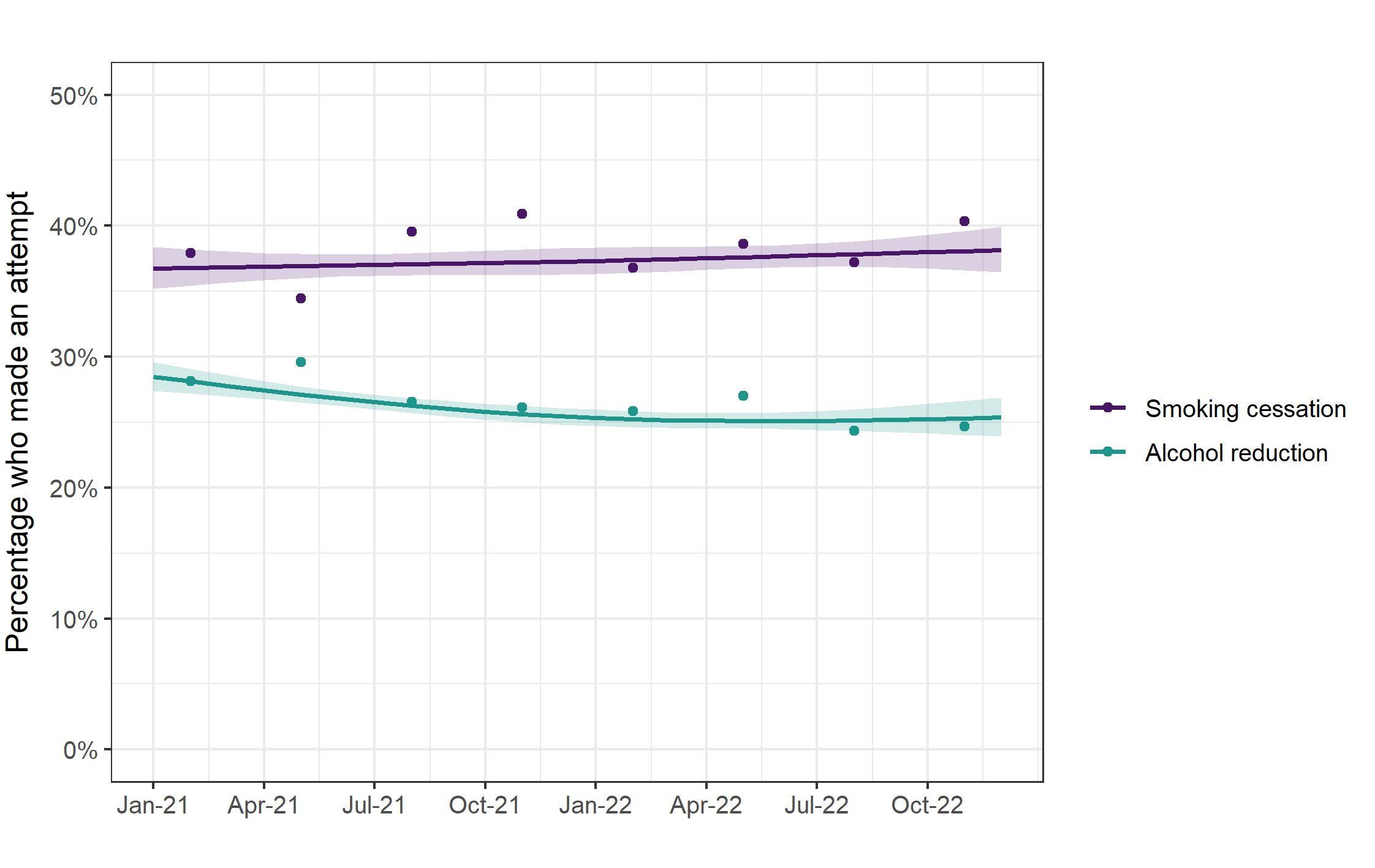

Supplement: S2 Fig — Lines represent modelled weighted prevalence by survey month, modelled non-linearly using restricted cubic splines (three knots), adjusting for covariates. Shaded bands represent standard errors. Points represent raw weighted prevalence by quarter. (TIFF) [file pone.0286183.s002.tiff]

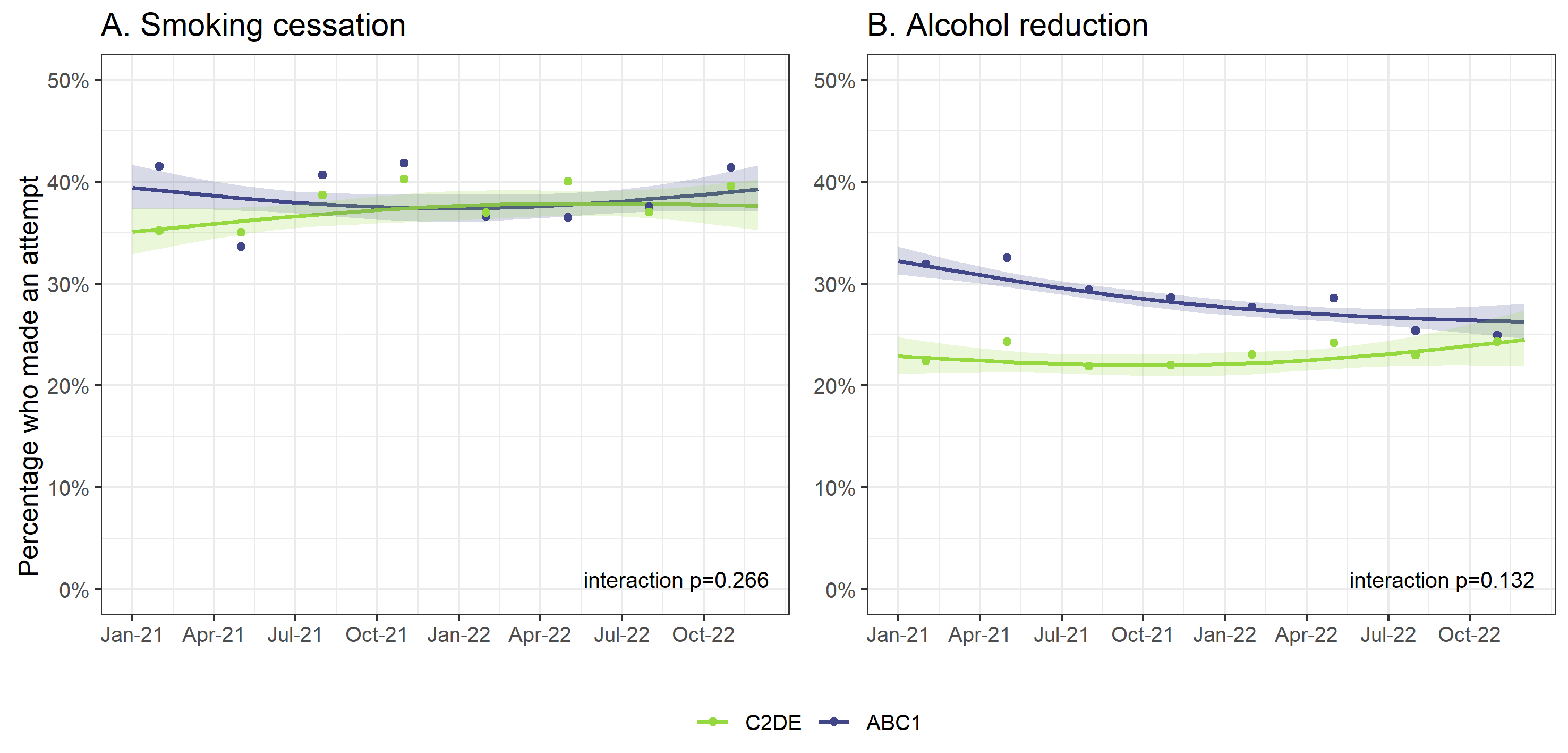

Supplement: S3 Fig — Lines represent modelled weighted prevalence by survey month, modelled non-linearly using restricted cubic splines (three knots), adjusting for covariates. Shaded bands represent standard errors. Points represent raw weighted prevalence by quarter. P-values are for the interaction between survey month and social grade. (TIFF) [file pone.0286183.s003.tiff]

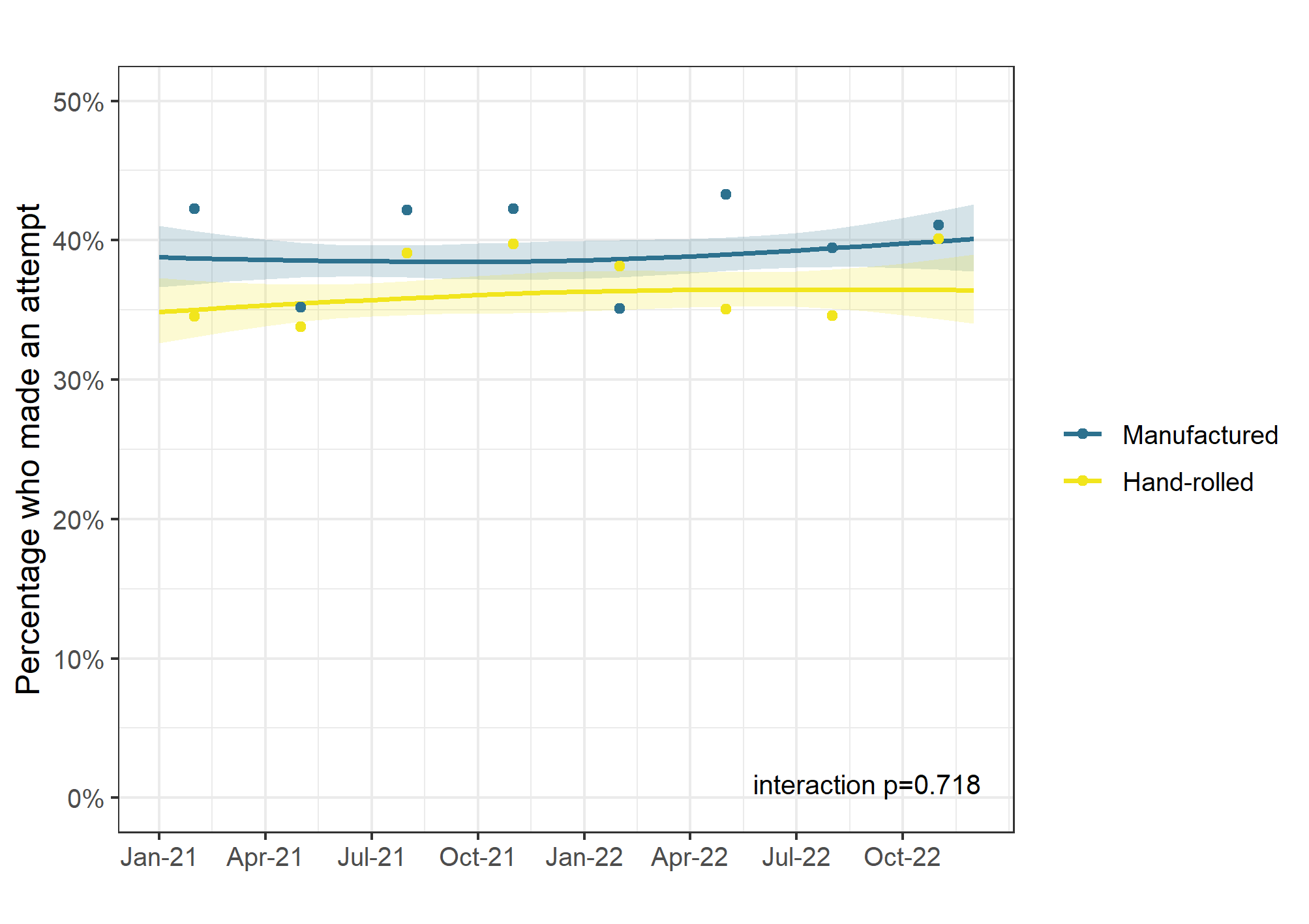

Supplement: S4 Fig — Lines represent modelled weighted prevalence by survey month, modelled non-linearly using restricted cubic splines (three knots), adjusting for covariates. Shaded bands represent standard errors. Points represent raw weighted prevalence by quarter. P-values are for the interaction between survey month and main type of cigarettes smoked. (TIFF) [file pone.0286183.s004.tiff]

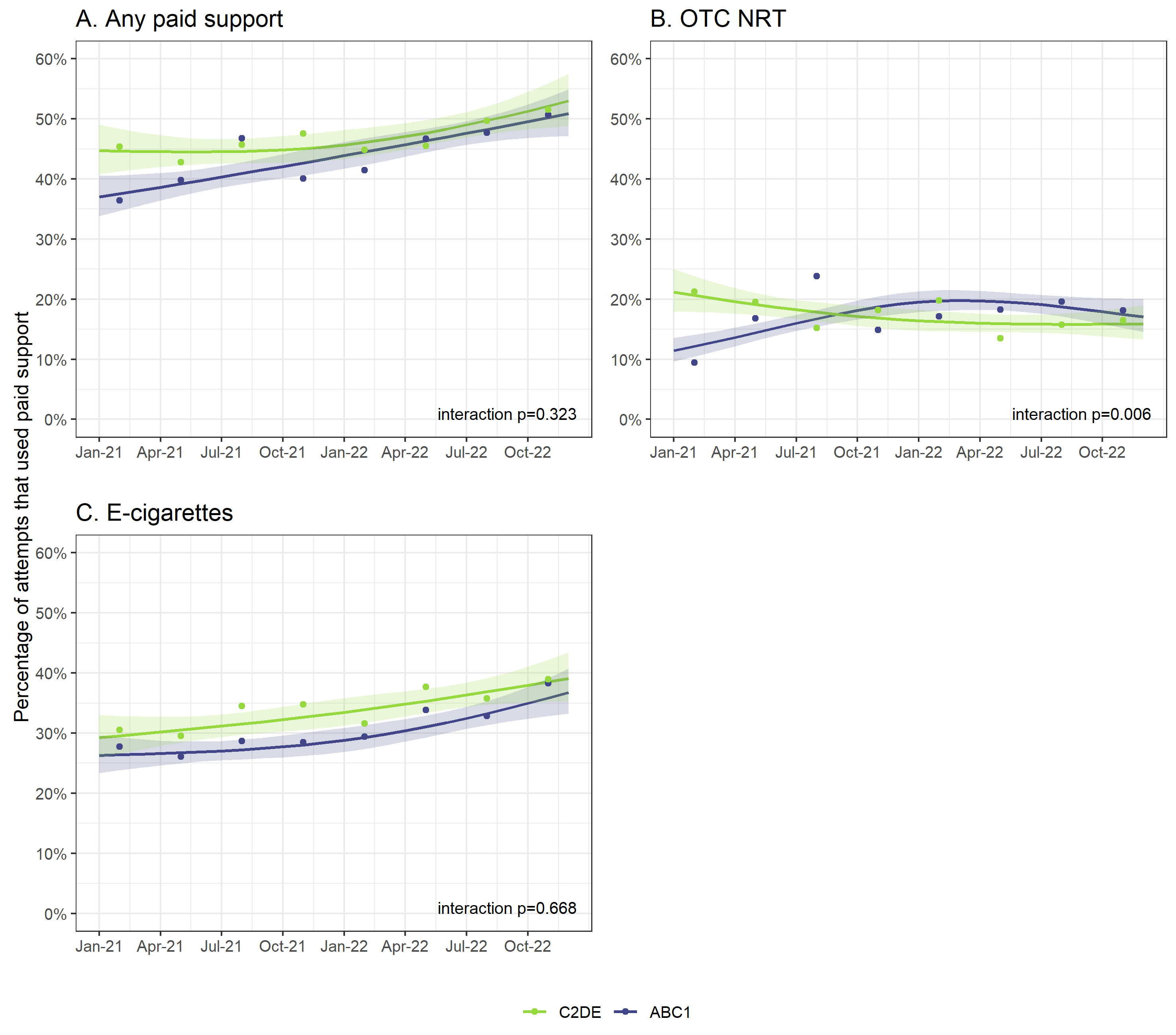

Supplement: S5 Fig — Lines represent modelled weighted prevalence by survey month, modelled non-linearly using restricted cubic splines (three knots), adjusting for covariates. Shaded bands represent standard errors. Points represent raw weighted prevalence by quarter. P-values are for the interaction between survey month and social grade. (TIFF) [file pone.0286183.s005.tiff]

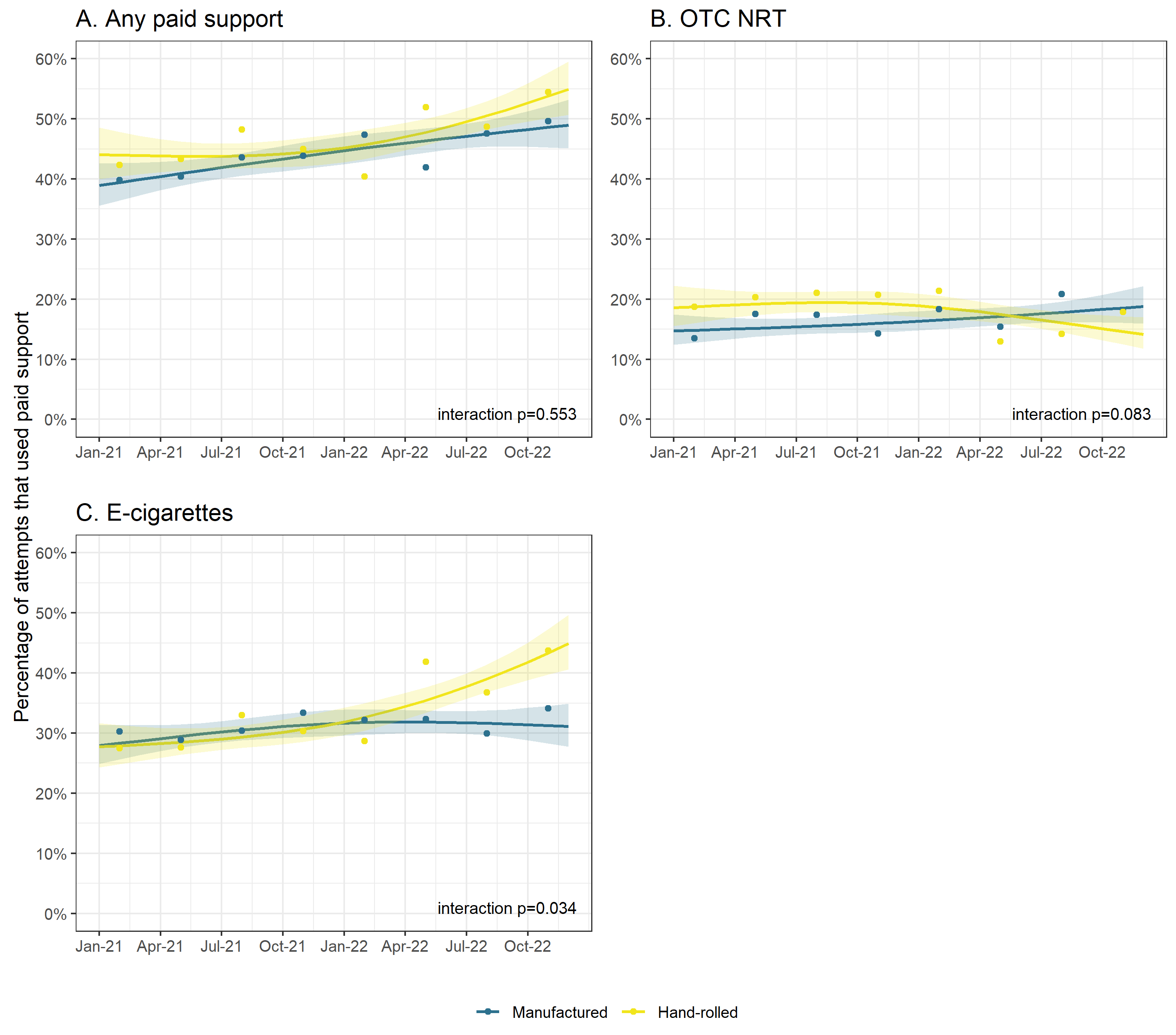

Supplement: S6 Fig — Lines represent modelled weighted prevalence by survey month, modelled non-linearly using restricted cubic splines (three knots), adjusting for covariates. Shaded bands represent standard errors. Points represent raw weighted prevalence by quarter. P-values are for the interaction between survey month and main type of cigarettes smoked. (TIFF) [file pone.0286183.s006.tiff]

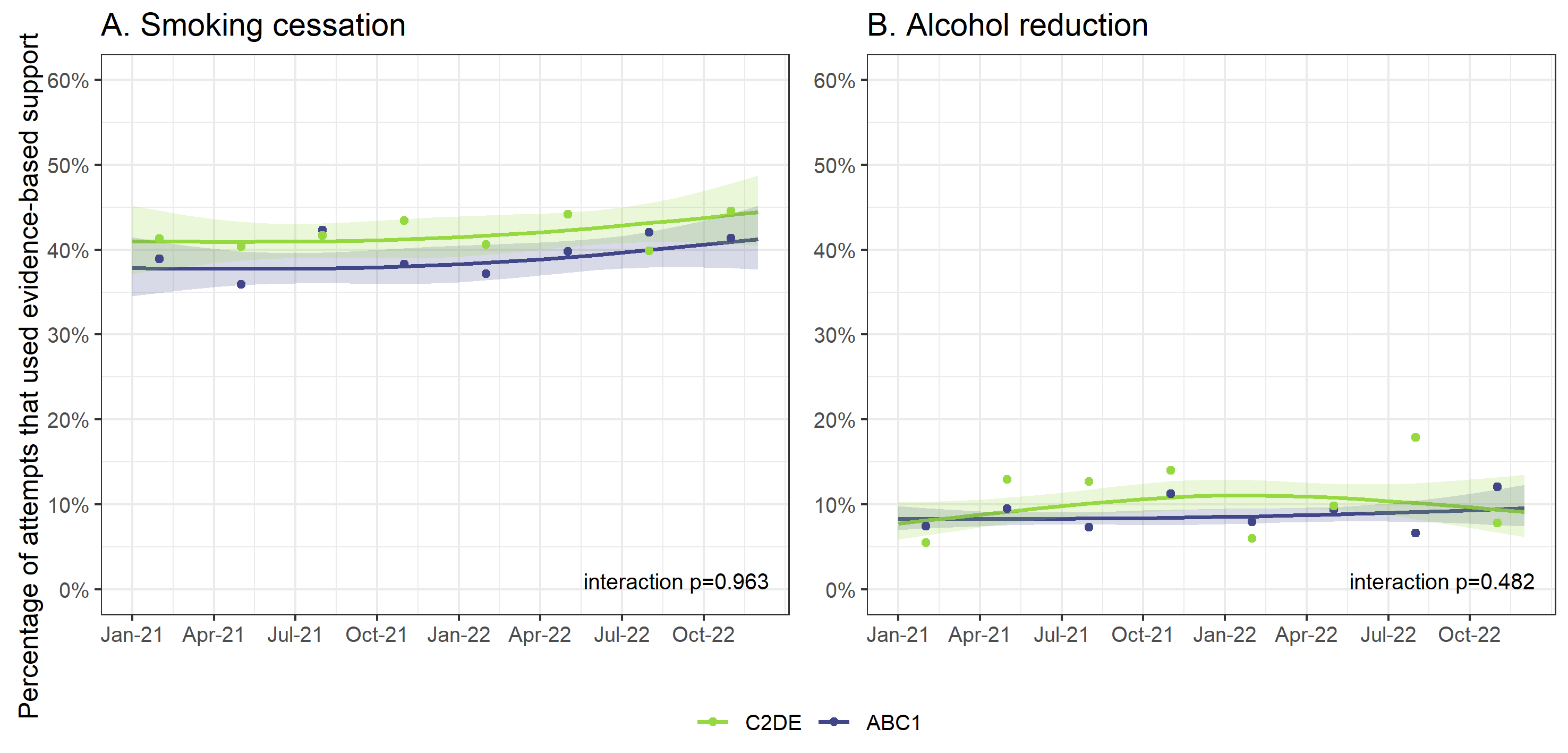

Supplement: S7 Fig — Lines represent modelled weighted prevalence by survey month, modelled non-linearly using restricted cubic splines (three knots), adjusting for covariates. Shaded bands represent standard errors. Points represent raw weighted prevalence by quarter. P-values are for the interaction between survey month and social grade. (TIFF) [file pone.0286183.s007.tiff]

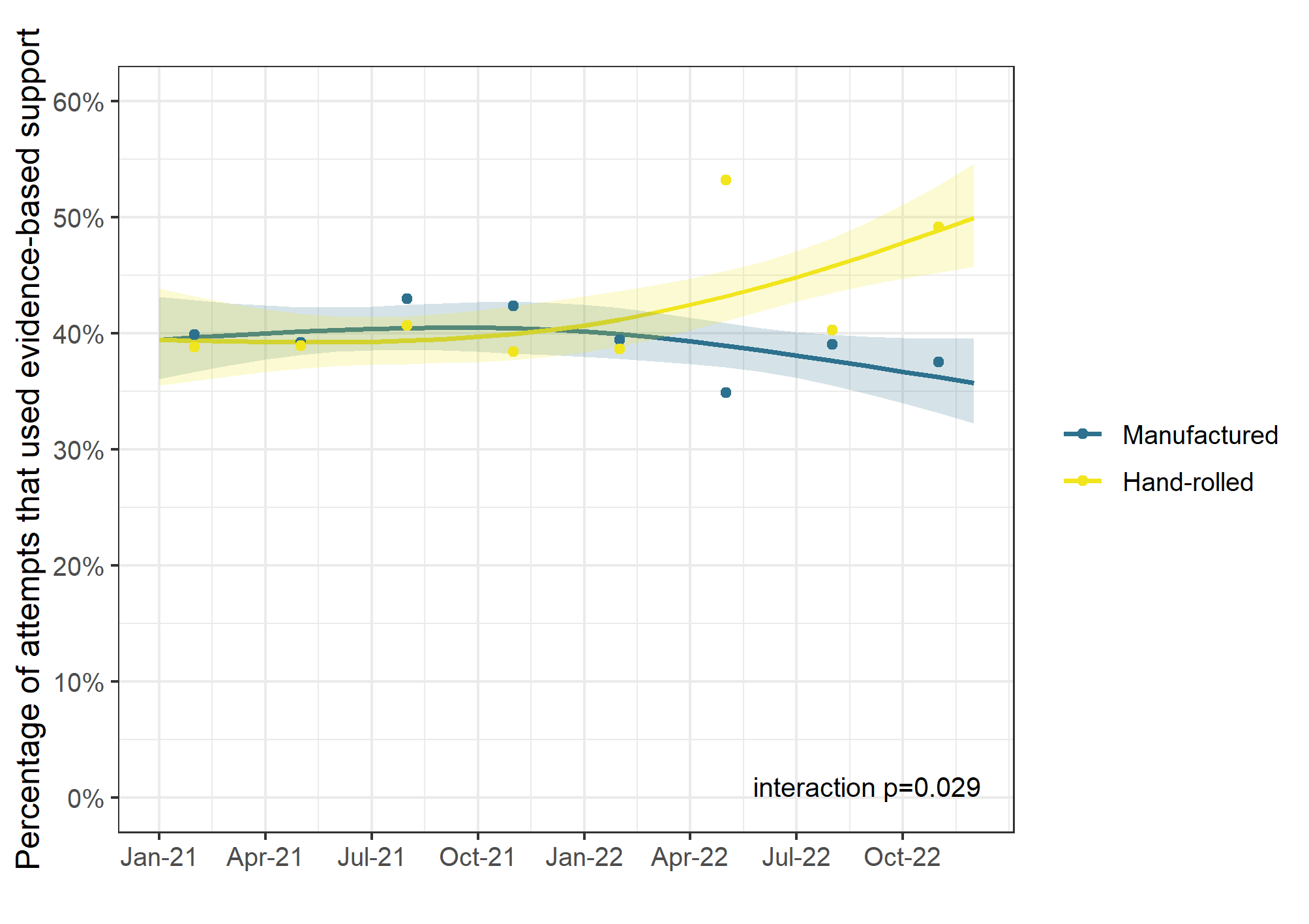

Supplement: S8 Fig — Lines represent modelled weighted prevalence by survey month, modelled non-linearly using restricted cubic splines (three knots), adjusting for covariates. Shaded bands represent standard errors. Points represent raw weighted prevalence by quarter. P-values are for the interaction between survey month and main type of cigarettes smoked. (TIFF) [file pone.0286183.s008.tiff]

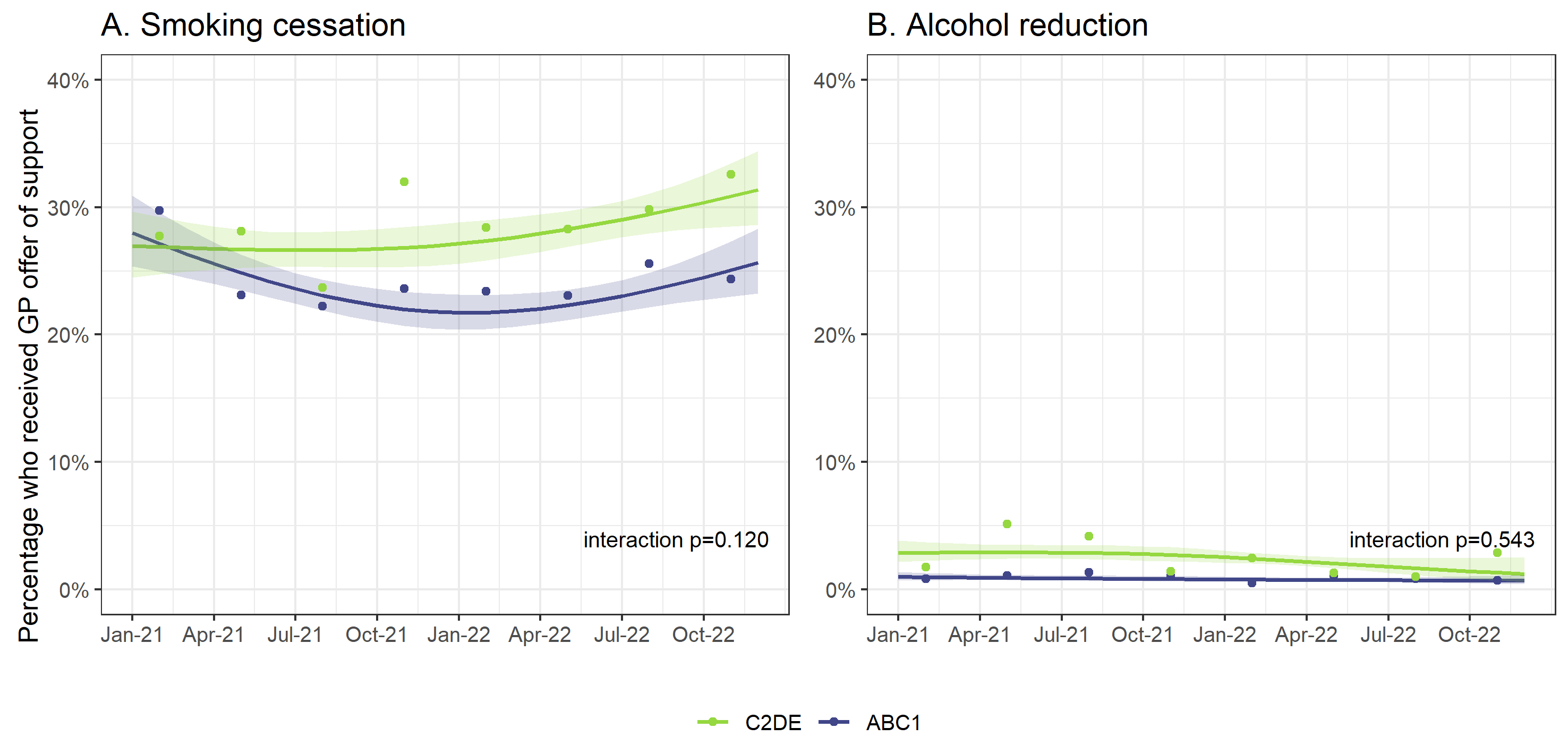

Supplement: S9 Fig — Lines represent modelled weighted prevalence by survey month, modelled non-linearly using restricted cubic splines (three knots), adjusting for covariates. Shaded bands represent standard errors. Points represent raw weighted prevalence by quarter. P-values are for the interaction between survey month and social grade. (TIFF) [file pone.0286183.s009.tiff]
