# Supplementary material for: Impact of the cost-of-living crisis on the nature of attempts to stop smoking and to reduce alcohol consumption in Great Britain: A representative population survey, 2021–2022
Source: PLoS One. 2023 May 23;18(5):e0286183. doi: 10.1371/journal.pone.0286183 (PMC10204963; doi:10.1371/journal.pone.0286183)
Supplement: Attachment — Submitted filename: Response v2.docx [file pone.0286183.s012.docx]

Journal Requirements:

Please review your reference list to ensure that it is complete and correct. If you have cited papers that have been retracted, please include the rationale for doing so in the manuscript text, or remove these references and replace them with relevant current references. Any changes to the reference list should be mentioned in the rebuttal letter that accompanies your revised manuscript. If you need to cite a retracted article, indicate the article’s retracted status in the References list and also include a citation and full reference for the retraction notice.
Response: We have checked our reference list and confirm it is complete and correct.

Additional Editor Comments:

Thanks for incorporating partly suggestions by the reviewers. I think the expenditure on smoking and its reduction due to Cost of living crisis is an important issue which you have not covered. Also we know price-elasticity or income-elasticity could be a determinantal components under cost-of-living crisis. Basic economic theory and consumer behaviour also highlights principles the substitution effects, i.e. if income reduces then consumer tends to reduce consumption of the particular good or move to a cheaper options (e.g. brom branded alcohol to local supermarket branded ones, same is the case for smoking). Your paper failed to address this behaviour. My suggestions are to provide a detailed limitations of your study section by answering what factors are supported from the data and what aspects are not covered due to lack of information in the dataset.
Response: Thank you for raising these issues. We have expanded our limitations section to address them:

“Thirdly, information on product choice (e.g., brands purchased) was not captured by the survey, so we were unable to determine the extent to which people mitigated cost pressures by switching to cheaper products (e.g., from branded alcohol to supermarket own-brands). In addition, we did not analyse changes in cigarette consumption so our data do not offer any insight into whether people are smoking fewer cigarettes in order to reduce their expenditure on tobacco during the cost-of-living crisis.”s

Reviewers' comments:
[None]
